# Identification of *TNFRSF1A* as a novel regulator of carfilzomib resistance in multiple myeloma

**DOI:** 10.32604/or.2023.030770

**Published:** 2023-12-28

**Authors:** JIE ZHAO, XUANTAO YANG, HAIXI ZHANG, XUEZHONG GU

**Affiliations:** 1 Department of Hematology, The First People’s Hospital of Yunnan Province, Yunnan Province Clinical Research Center for Hematologic Disease, Yunnan Province Clinical Center for Hematologic Disease, Kunming, 650032, China; 2 Department of Pathology, The First People’s Hospital of Yunnan Province, Kunming, 650032, China

**Keywords:** Multiple myeloma, Carfilzomib, Drug resistance, Major histocompatibility complex, *TNFRSF1A*

## Abstract

Multiple myeloma (MM) is a hematological tumor with high mortality and recurrence rate. Carfilzomib is a new-generation proteasome inhibitor that is used as the first-line therapy for MM. However, the development of drug resistance is a pervasive obstacle to treating MM. Therefore, elucidating the drug resistance mechanisms is conducive to the formulation of novel therapeutic therapies. To elucidate the mechanisms of carfilzomib resistance, we retrieved the GSE78069 microarray dataset containing carfilzomib-resistant LP-1 MM cells and parental MM cells. Differential gene expression analyses revealed major alterations in the major histocompatibility complex (MHC) and cell adhesion molecules. The upregulation of the tumor necrosis factor (TNF) receptor superfamily member 1A (*TNFRSF1A*) gene was accompanied by the downregulation of MHC genes and cell adhesion molecules. Furthermore, to investigate the roles of these genes, we established a carfilzomib-resistant cell model and observed that carfilzomib resistance induced *TNFRSF1A* overexpression and *TNFRSF1A* silencing reversed carfilzomib resistance and reactivated the expression of cell adhesion molecules. Furthermore, *TNFRSF1A* silencing suppressed the tumorigenesis of MM cells in immunocompetent mice, indicating that *TNFRSF1A* may lead to carfilzomib resistance by dampening antitumor immunity. Furthermore, our results indicated that *TNFRSF1A* overexpression conferred carfilzomib resistance in MM cells and suppressed the expression of MHC genes and cell adhesion molecules. The suppression of MHC genes and cell adhesion molecules may impair the interaction between immune cells and cancer cells to impair antitumor immunity. Future studies are warranted to further investigate the signaling pathway underlying the regulatory role of *TNFRSF1A* in MM cells.

## Introduction

Multiple myeloma (MM) is the most common hematological tumor and cancer recurrence is a common challenge in the clinical management of MM [1]. An unhealthy lifestyle, including chronic drinking and smoking, and genetic factors are common risk factors for the initiation of MM [2–4]. Chemotherapeutic drugs targeting different cellular processes, including dexamethasone, lenalidomide, doxorubicin, cyclophosphamide, and vincristine, have been approved to treat MM. However, the development of drug resistance has been found in almost all clinical drugs [5–7].

Drugs with defined modes of action have provided novel strategies for cancer treatment, especially those with the accessibility of next-generation sequencing technology to dissect the dysregulation of specific cellular targets [8]. Bortezomib was developed as the first-generation proteasome inhibitor for treating MM. This drug causes endoplasmic reticulum (ER) stress due to the accumulation of unfolded proteins [9]. The unfolded protein response leads to apoptosis by activating pro-apoptotic signaling and cellular oxidative stress. Carfilzomib was approved by the US Food and Drug Administration (FDA) as the second-generation proteasome inhibitor for treating refractory MM, which exerted a superior effect compared with bortezomib [10,11]. The combined administration of carfilzomib and dexamethasone can prolong the overall survival of patients with MM compared with the combination of bortezomib and dexamethasone in phase III clinical trials [12]. Unfortunately, similar to other targeted therapies, the occurrence of drug resistance to proteasome inhibitors is an inevitable event in the rapidly evolving tumor cell population [13]. Ongoing efforts are attempting to investigate the genetic factors driving carfilzomib resistance in MM. Even though multiple hypotheses, including proteasomal adaptations and the upregulation of multidrug resistance protein, explain the development of carfilzomib resistance [13–15], the exact mechanisms are yet to be elucidated.

Recent evidence has suggested the immunomodulatory activity of carfilzomib in treating MM. A combination of carfilzomib and immunomodulatory drugs exhibited promising effects in prolonging the survival of newly diagnosed patients with MM [16]. Nevertheless, immune escape leads to the progression of myeloma, including changes in the bone marrow immune microenvironment [17]. This is associated with the changes in many immune cells, such as macrophages, dendritic cells, T cells, and natural killer cells. Myeloma cells can also undergo drastic reprogramming to attenuate antigen presentation and escape immune surveillance [18]. However, whether carfilzomib resistance also contributes to immune escape in MM has not been reported.

In this study, we investigated the gene candidates contributing to carfilzomib resistance in MM using a published dataset (GSE78069), which contained the analyses of the carfilzomib-resistant LP-1 MM cell line and the parental LP-1 cell line. Through differential gene expression analysis and protein–protein interaction network analysis, we found that *TNFRSF1A* is the key candidate that is upregulated in carfilzomib-resistant LP-1 MM cells. We also established a carfilzomib-resistant MM cell model and investigated the functional role of *TNFRSF1A* in causing carfilzomib sensitivity in *in vitro* and *in vivo* models.

## Materials and Methods

### Public data retrieval

The microarray data of carfilzomib-resistant and parental LP-1 human MM cells were retrieved from the Gene Expression Omnibus (GEO) dataset GSE78069. The LP-1 cells were exposed to increasing doses of carfilzomib for 18 weeks. Cells adapted to growth in 4 nM carfilzomib for 4 weeks, in 6 nM in the next 6 weeks, and in 12 nM for a further 8 weeks. The microarray analysis was performed using carfilzomib-resistant cells in the absence of carfilzomib for one week and in the parental LP-1 cells. Three biological replicates were performed for each type of cell line.

### Differential gene expression analysis

Data were downloaded from the GEO database by the GEO query package, redundant probes were removed and the differences between the two groups were analyzed using the Limma package. Gene IDs were converted by the clusterProfiler package, and ggplot2 (version 3.3.3) was used for visualization analysis. The differentially expressed genes (DEGs) were defined as the |log2 (Fold Change)| > 0.5 and the adjusted *p*-value was < 0.05.

### Gene ontology (GO) and kyoto encyclopedia of genes and genomes (KEGG) analyses

ClusterProfiler package (version 3.14.3) was used for performing enrichment analysis and org.rn.g.db package [version 3.10.0] was used for ID conversion. The ggplot2 package of R software (version 4.2.2) was used for performing statistical analysis and visualization.

### Protein–protein interaction network

The STRING website was used to obtain the PPI network of common differentially expressed genes and the table of interaction relationships among differentially expressed genes was obtained. Cytoscape was used to identify and establish the PPI network. We used Cytohubba and mcode for hub gene recognition, which are the built-in modules of Cytoscape.

### Cell culture

Murine myeloma cell line MPC-11 and bone marrow stromal HS-5 cells were purchased from Cobioer Biosciences (Nanjing, Jiangsu, China). The cell lines were authenticated by the supplier using the STR profiling method. The cells were maintained in McCoy’s medium (Thermo Fisher Scientific, Waltham, MA, USA) containing 10% fetal bovine serum (Thermo Fisher Scientific, Waltham) and 1% penicillin/streptomycin (Hyclone, Logan, UT, USA) under the condition of 37°C and 5% CO_2_. To establish the carfilzomib-resistant MPC-11 cells, the cells were exposed to the following increasing dose of carfilzomib (MedChemExpress, Shanghai, China): 4 nM carfilzomib for 4 weeks, 6 nM for the next 6 weeks, and 12 nM for further 8 weeks. The experiments were then performed between the resistant cells and the parental cell line. The following experiment was performed by treating both cells with 4 nM carfilzomib.

For the cell co-culture experiment, HS-5 cells were pre-labeled with CellTrace™ Violet dye (Thermo Fisher Scientific) to differentiate them from MPC-11 cells during flow cytometry analysis. MPC-11 and HS-5 cells were seeded at a 1:1 ratio (2 × 10^5^ cells/well in a 6-well plate) and co-cultured for 48 h in the presence of 4 nM carfilzomib. Tumor necrosis factor (TNF)-α protein was purchased from Genscript Biotech company (Nanjing, Jiangsu, China), and added at 10 ng/mL for 48 h.

### Lentivirus-mediated TNFRSF1A silencing

The lentivirus carrying a scramble shRNA or shRNAs targeting *TNFRSF1A* was produced by Genscript Biotech company. To generate stable cells with *TNFRSF1A* knockdown, MPC-11 cells were inoculated in a 6-well plate at 2.5 × 10^5^ cells/well. The cells were infected with the corresponding recombinant lentivirus at a multiplicity of infection (MOI) of 5 in the presence of 10 µg/mL polybrene (Sigma, Shanghai, China). Infected cells were selected using 1.0 µg/mL puromycin (Sigma) for 2 weeks to eliminate the uninfected cells before further experiments.

### Cell counting kit-8 (CCK-8) assay

MPC-11 cells were seeded into a 96-well plate at a density of 1500 cells/well and cultured in a humidified cell culture incubator for 0, 24, 48, and 72 h in the presence of 4 nM carfilzomib. Subsequently, 10 μL of CCK-8 reaction solution (Solarbio, Beijing, China) was added to the cell culture at indicated time points and incubated for 3 h in the humidified cell culture incubator. The light absorption value (OD value) of each condition was measured at a wavelength of 450 nm using the Synergy H1 microplate reader (Winooski, Vermont, CA, USA).

### Apoptosis detection using flow cytometry

Cell apoptosis was detected using the FITC Annexin V Apoptosis Detection Kit (BD Biosciences, San Jose, CA, USA) according to the manufacturer’s protocols. Briefly, 5 µL of annexin V-FITC and PI reagent were added to 1 mL of the cell suspension with 1 × 10^6^ cells. The cells were stained for 30 min in the dark, and the stained cells were washed twice with 1× phosphate-buffered saline (PBS). After washing, the cells were resuspended in 400 µL of PBS. The percentage of apoptotic cells was detected using the BD FACS CantoTM II Flow Cytometer (BD Biosciences).

### Real-time quantitative reverse transcription PCR (RT-qPCR) assay

TRIzol reagent (Thermo Fisher Scientific, Waltham, MA, USA) was used to extract RNA from tissues and cells according to the protocol. Then, one µg of total RNA was used for reverse transcription using the RevertAid First Strand cDNA Synthesis Kit (Thermo Fisher Scientific). The resulting cDNA was analyzed using the 7500 Real-Time PCR System (Applied Biosystems, Carlsbad, CA, USA), using SYBR premix EX TAQ II kit (Takara, Dalian, Jilin, China). The PCR cycling conditions were as follows: 95°C for 2 mins, 40 cycles of 95°C for 30 s, 60°C for 30 s, and 72°C for 60 s. The 2^−∆∆Ct^ method was performed to analyze the relative expression, and actin was used as the internal reference gene. All primer sequences were synthesized and purchased from Sangon Biotechnology Co., Ltd. (Shanghai, China).

### Western blotting analysis

Total protein was extracted from cells using RIPA lysis buffer containing protease inhibitor cocktail (Beyotime Biotechnology, Beijing, China). The supernatant containing total protein lysate was quantified using a BCA protein assay kit (Beyotime Biotechnology). A total of 10 µg of protein was used for sodium dodecyl sulfate–polyacrylamide gel electrophoresis. The separated proteins were transferred onto a polyvinylidene difluoride membrane. After blocking with 5% skimmed milk for 1 h, the membrane was incubated with primary antibodies (1:1000 dilution, all from Abcam, Cambridge, UK) overnight at 4°C. The following antibodies were used: *TNFRSF1A* (ab194814), CXCL10 (ab306587), Sell (CD62L) (ab264045), ICAM1 (ab2213), NCAM1 (ab9272), and beta-actin (ab6276). The membrane was washed thrice using 1× Tris-Buffered Saline and 0.1% Tween® 20 Detergent (TBST) buffer. The membrane was further incubated with a horseradish peroxidase (HRP)-linked secondary antibody (1:3000; Cell signaling technology, #7074, MA, USA) at room temperature for 1 h. The protein bands were developed using an enhanced chemiluminescence kit (Santa Cruz, TX, USA) and photographed on a gel imager system (Bio-Rad, Hercules, CA, USA).

### Animal model

All animal experiments were approved by the animal care and use ethical committee of the First People’s Hospital of Yunnan Province (Approval number: KHLL2022-SQ111). Twenty-four male Balb/c mice weighing 30–40 g at 3–4 weeks old were housed in specific pathogen-free conditions on a 12-h light/dark cycle with free access to food and water. The mice were randomly divided into the following four groups (6 mice in each group): (1) MM group (injected with parental MPC-11), (2) MM/carfilzomib group (injected with carfilzomib-resistant MPC-11 cells), (3) MM/carfilzomib + Lenti-sh-NC group (injected with carfilzomib-resistant MPC-11 cells infected with the scramble shRNA), and (4) MM/carfilzomib+lenti-sh-*TNFRSF1A* group (injected with carfilzomib-resistant MPC-11 cells infected with *TNFRSF1A* shRNAs). Then, 200 μL of the cell suspension (1 × 10^6^ cells) was injected into the flank of each mouse. All the mice were injected with the following carfilzomib injection method: 3 mg/kg by intravenous infusion for two consecutive days per week within three weeks (i.e., days 1, 2, 8, 9, 15, and 16), followed by 12 days of rest (days 17–28). Tumor volume was monitored every week post-injection. The tumors were monitored using a caliper weekly and the tumor volume was calculated using the formula: V (tumor) = 0.5 × length × width^2^. If the tumor xenograft exceeded 2,000 mm^3^, the mice were euthanized immediately. Four weeks after tumor cell inoculation, all the mice were euthanized via CO_2_ asphyxiation, and death was assured by subsequent cervical dislocation. The tumors of terminally dead mice were resected for weight measurement and terminal deoxynucleotidyl transferase-mediated dUTP-biotin nick end labeling (TUNEL) assay.

### TUNEL

TUNEL cell apoptosis detection kit (Beyotime Biotechnology) was used for cell death staining in the tissue sections. Then, 5-μm sections of formalin-fixed paraffin-embedded tumor tissue were deparaffinized and rehydrated. The sections were incubated with 20 µg/mL Protease K without DNase at 37°C for 15 min. After washing with PBS, the sections were incubated in 3% hydrogen peroxide solution at 22°C for 10 min and then labeled with the biotin labeling solution containing TdT enzyme (2 μL), Biotin-dUTP (48 μL), and biotin labeling solution (50 µL) at 37°C for 60 min. After washing, the section was further incubated with a streptavidin HRP working solution at room temperature for 30 min. During incubation, the surrounding was kept moist with paper or cotton wool soaked with sufficient water to minimize the evaporation of the streptavidin HRP working solution. The signal development was performed using 200 µL of DAB color-developing solution at room temperature for 5–30 min, and the nuclei were stained using a hematoxylin staining solution. The sections were dehydrated using 95% ethanol for 5 min and treated with xylene twice for 5 min before observation.

### Statistical analysis

Statistical analyses were performed using GraphPad Prism version 9.0. (GraphPad, San Diego, CA, USA). The statistical difference between the two groups was compared using an unpaired Student’s *t* test. Comparisons among multiple groups were analyzed using one-way analysis of variance with Tukey’s *post-hoc* test for pairwise comparison. Data are presented as the mean ± standard deviation (SD). A *p*-value of < 0.05 was considered statistically significant.

## Results

### Differential gene expression analysis of carfilzomib-resistant and sensitive MM cells

**Figure 1:**
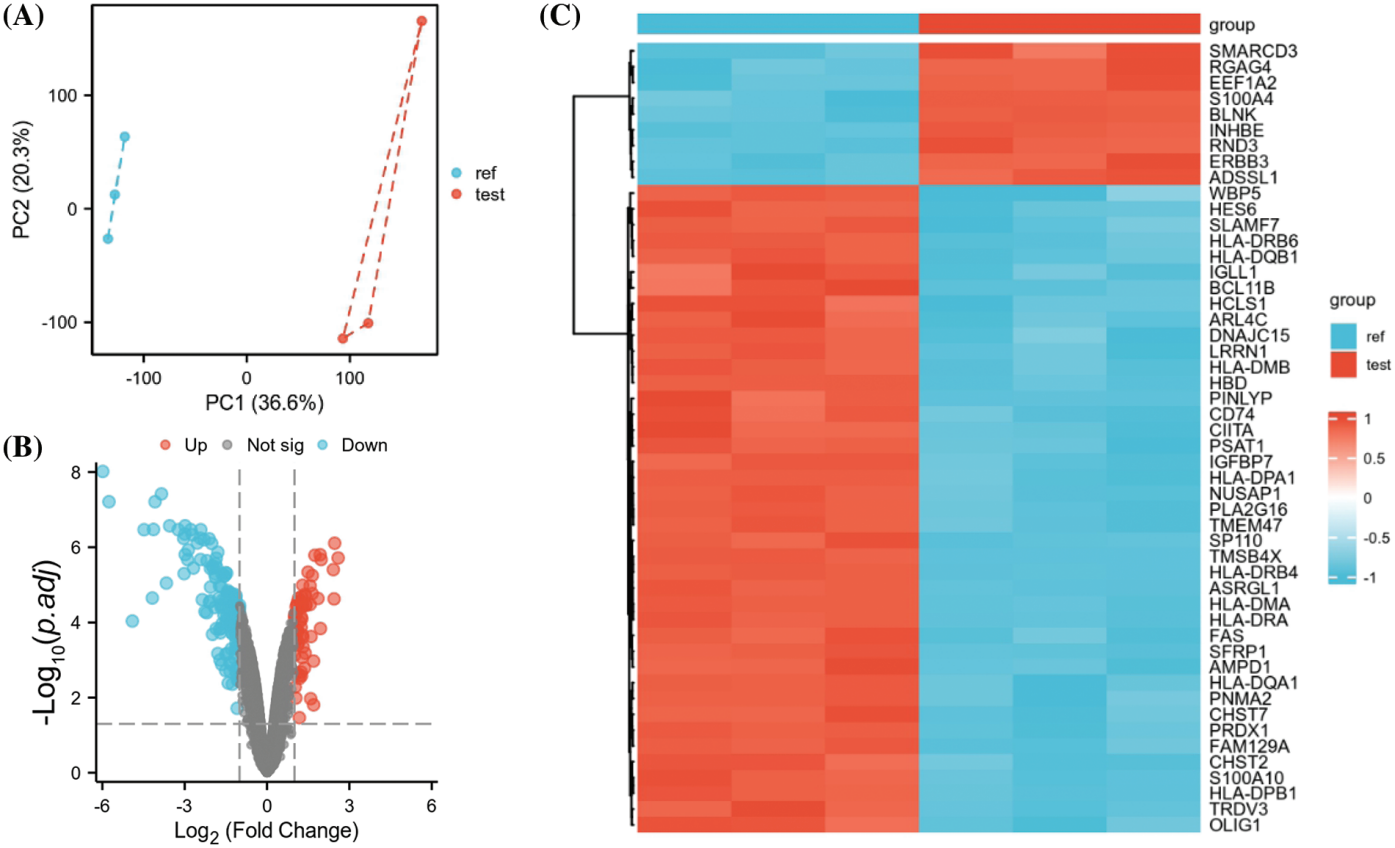
GEO data set GSE78069 was used for differential gene expression analysis between carfilzomib-resistant LP-1 cells and untreated LP-1 cells. (A) Principal component analysis of the transcriptomes of carfilzomib-resistant LP-1 cells (test) and untreated LP-1 cells (ref). (B) Volcano plot showing the differentially expressed genes (DEGs), with |log2 (FC) | > 0.5 and the adjusted *p*-value < 0.05. (C) The heat map displays the top 50 DEGs.

The GEO dataset GSE78069 was used to elucidate differential gene expression in carfilzomib-resistant and sensitive MM cells. The dataset comprises three carfilzomib-resistant LP-1 cell samples and three parental cell samples. Principle component analysis revealed good separation of carfilzomib-resistant (test) and parental cell samples (ref), suggesting significant changes in the transcriptomes (Fig. 1A). The volcano plot revealed 923 DEGs; 404 were upregulated and 519 were downregulated (Fig. 1B). The heat map in Fig. 1C presents the top 50 DEGs; most genes were downregulated in carfilzomib-resistant cells.

**Figure 2:**
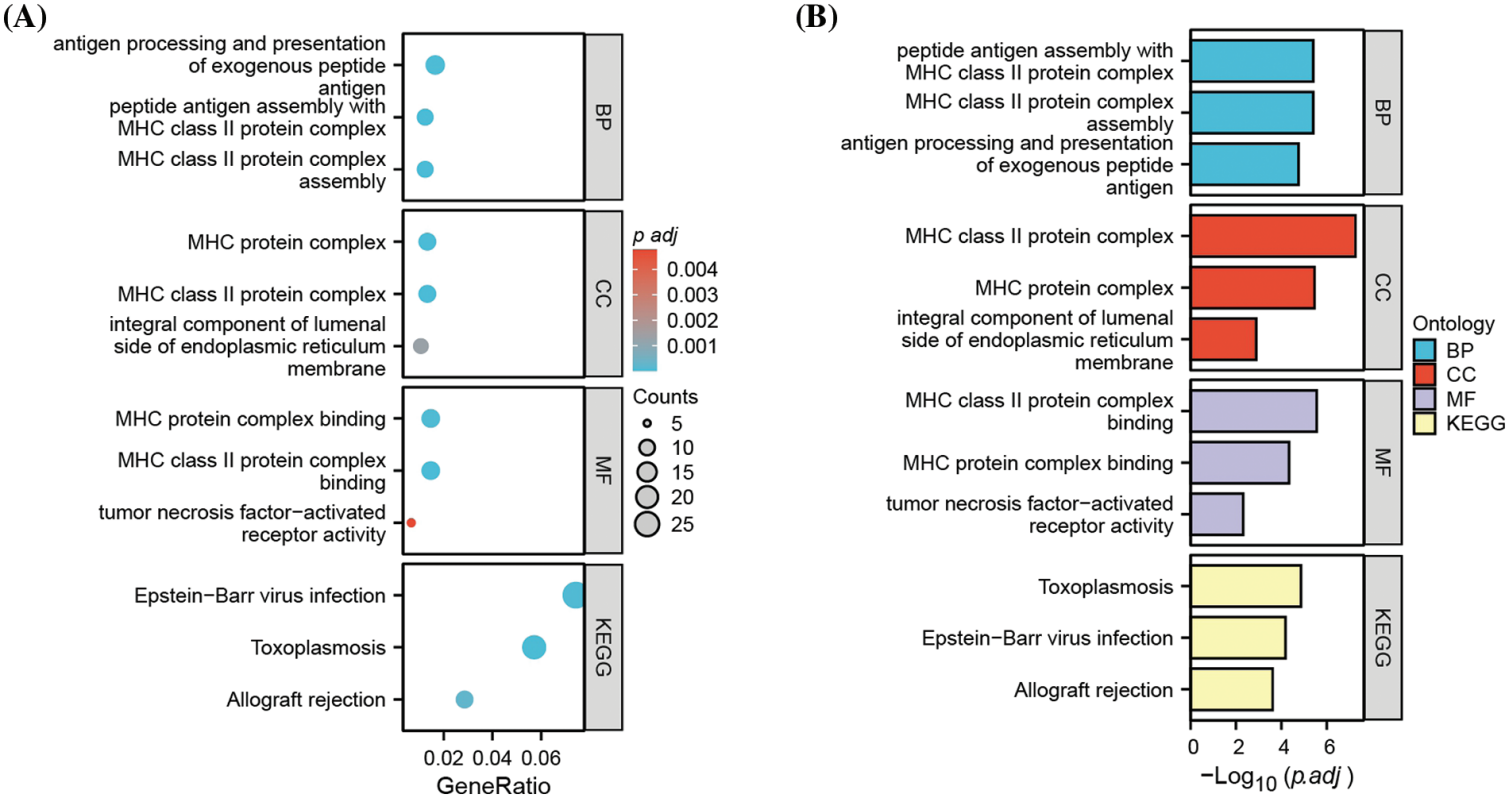
Functional enrichment analysis of DEGs. (A) Gene ontology (GO) and KEGG pathway enrichment analysis of DEGs, showing the gene counts and adjusted *p* value in each term. (B) Gene ontology (GO) and KEGG pathway enrichment analysis of DEGs, showing the −Log10 of adjusted *p* value in each term. BP: biological processes; CC: cellular components; MF: molecular function.

To obtain additional biological insights into the identified DEGs, GO and KEGG pathway enrichment analyses were performed. The most significantly enriched biological processes, cellular components, and molecular functions were antigen processing and presentation, major histocompatibility complex (MHC), and MHC protein complex binding (Fig. 2); this suggests that MHC-mediated antigen presentation is the predominant cellular process altered after MM cells acquire carfilzomib resistance.

### PPI network analysis reveals key regulatory modules involved in antigen presentation

**Figure 3:**
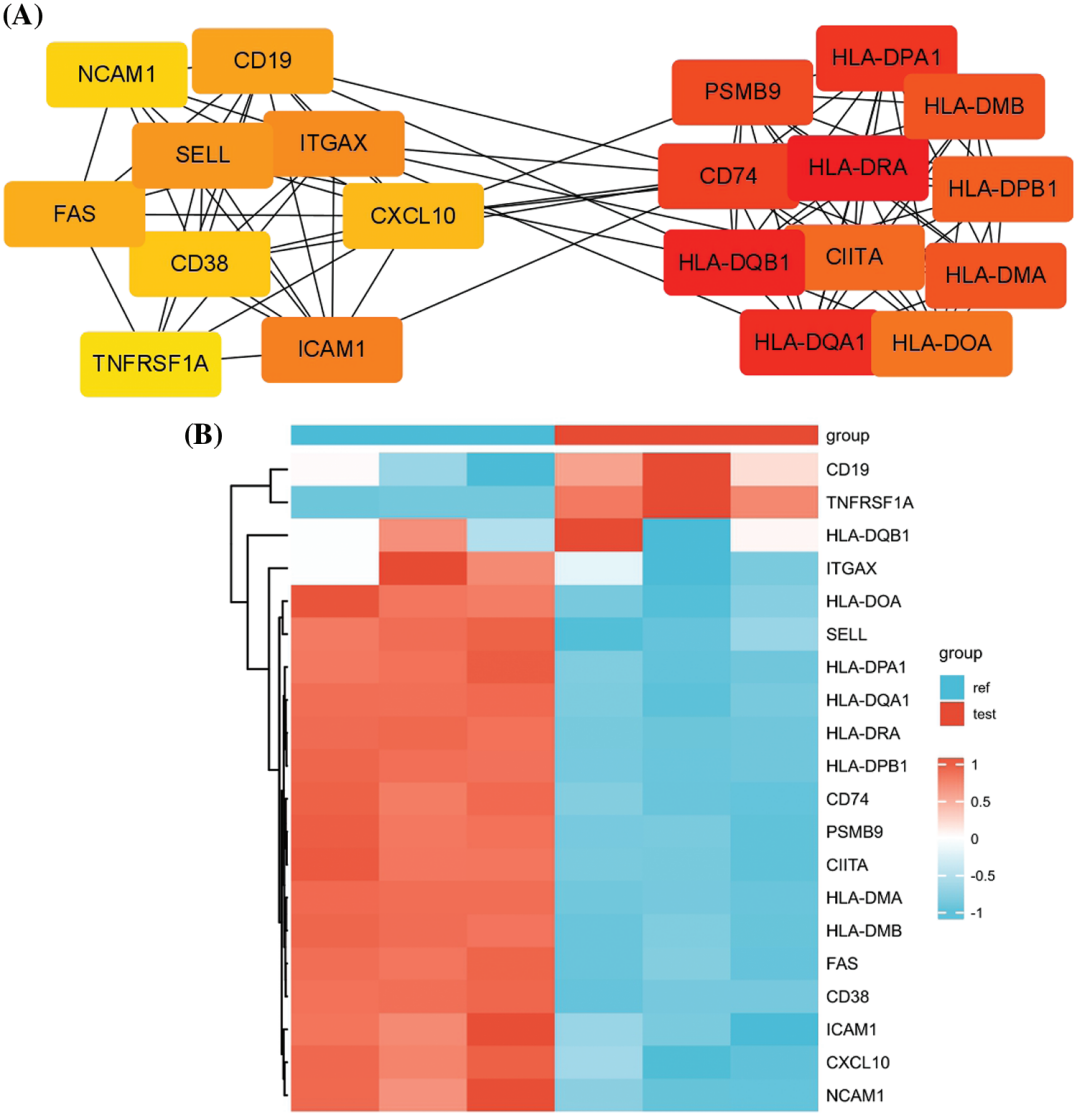
PPI network analysis of the DEGs. (A) PPI analysis by retrieving the protein-protein interaction relationship from the STRING database. Cytohubba and mcode modules of Cytoscape were used for hubgene identification. (B) The heat map displays the relative expression levels of hubgenes in carfilzomib-resistant LP-1 cells (test, Red group) and the parental LP-1 cells (ref, Blue group).

To identify the key regulatory modules in the DEGs, PPI network analysis was performed by retrieving the PPI relationships from the STRING database. Cytoscape analysis revealed two key interaction modules (Fig. 3A). The right network mainly comprised multiple components of the MHC proteins, which were highly correlated. On the other hand, the left network appeared to contain several cell adhesion molecules such as SELL, ICAM1, NCAM1, and ITGAX and surface signaling molecules such as *TNFRSF1A*, CD19, CD38, and FAS. Of note, CD19 and *TNFRSF1A* were significantly upregulation in carfilzomib-resistant LP-1 cells, whereas other genes were mostly downregulated (Fig. 3B). Because CD19 is a well-known marker for different types of myeloma clones and has been used as an immunotherapy target in MM treatment [19,20], we selected *TNFRSF1A* as the new candidate that may serve as a key regulator in the network; this gene was consistently upregulated in all three carfilzomib-resistant LP-1 cell samples (Fig. 3B).

### Carfilzomib resistance leads to TNFRSF1A overexpression and the downregulation of cell adhesion molecules and MHC components

**Figure 4:**
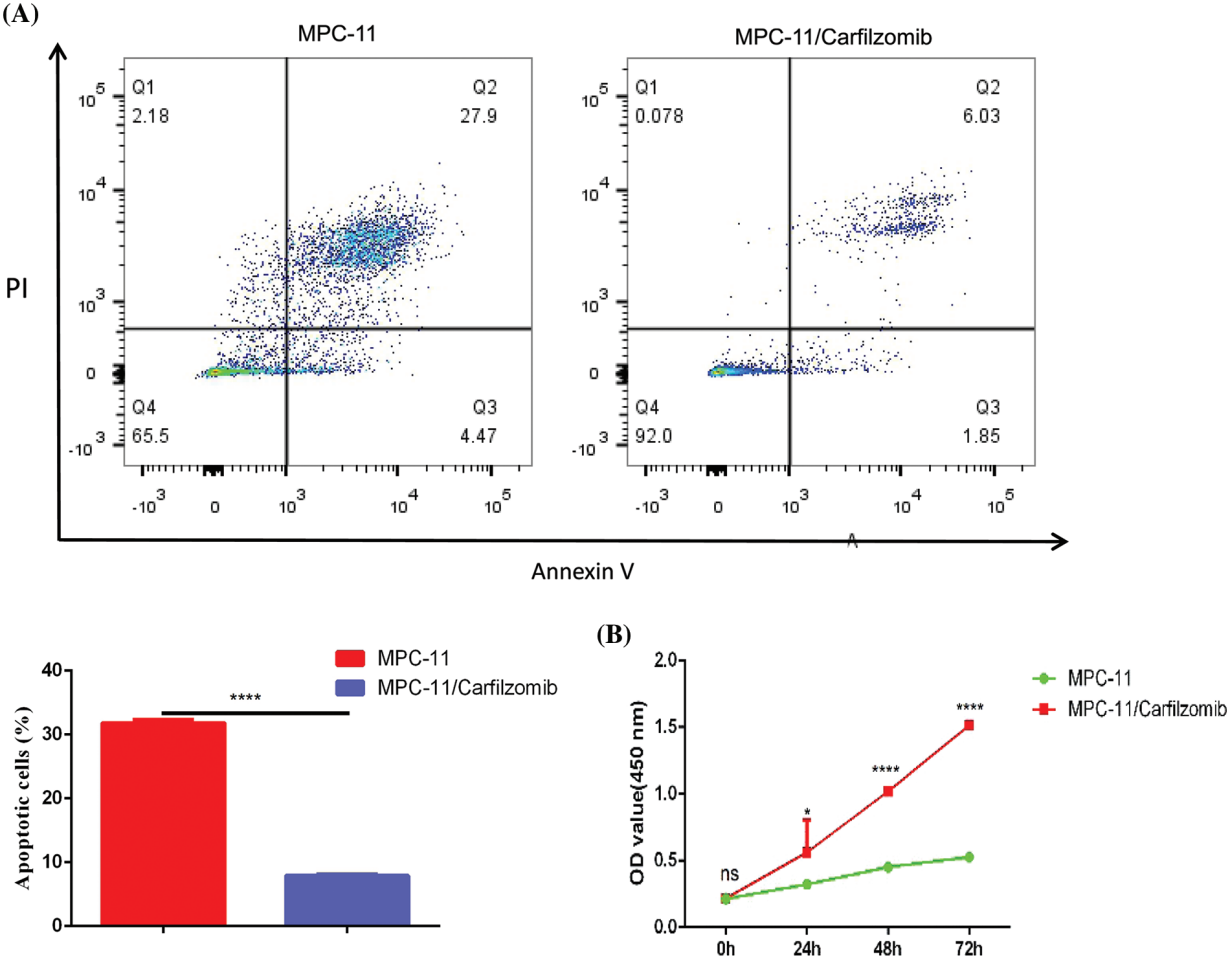
The establishment of carfilzomib-resistant MPC-11 cells. (A) Apoptosis detection by flow cytometry in carfilzomib-resistant MPC-11 cells and parental MPC-11 cells after the treatment with 4 nM carfilzomib for 48 h. (B) CCK-8 proliferation assay in carfilzomib-resistant MPC-11 cells and parental MPC-11 cells in the presence of 4 nM carfilzomib at different time points. Data are the summary of 3 independent experiments. *p < 0.05; ****p < 0.0001.

To confirm whether *TNFRSF1A* is involved in carfilzomib resistance of MM cells, we established the carfilzomib-resistant MM MPC-11 cell line (MPC-11/carfilzomib cells) by exposing the cells to increasing doses of carfilzomib. To verify carfilzomib resistance, MPC-11/carfilzomib cells and parental MPC-11 cells were treated with 4 nM carfilzomib for 48 h. Apoptosis detection via annexin V/PI staining revealed a significant decrease in the death of MPC-11/carfilzomib cells (Fig. 4A). Furthermore, the CCK-8 proliferation assay revealed the continuous proliferation of MPC-11/carfilzomib cells in the presence of 4 nM carfilzomib; in contrast, the proliferation of parental MPC-11 cells was suppressed by carfilzomib at different time points (Fig. 4B). Taken together, these findings confirm the development of carfilzomib resistance in MPC-11/carfilzomib cells.

**Figure 5:**
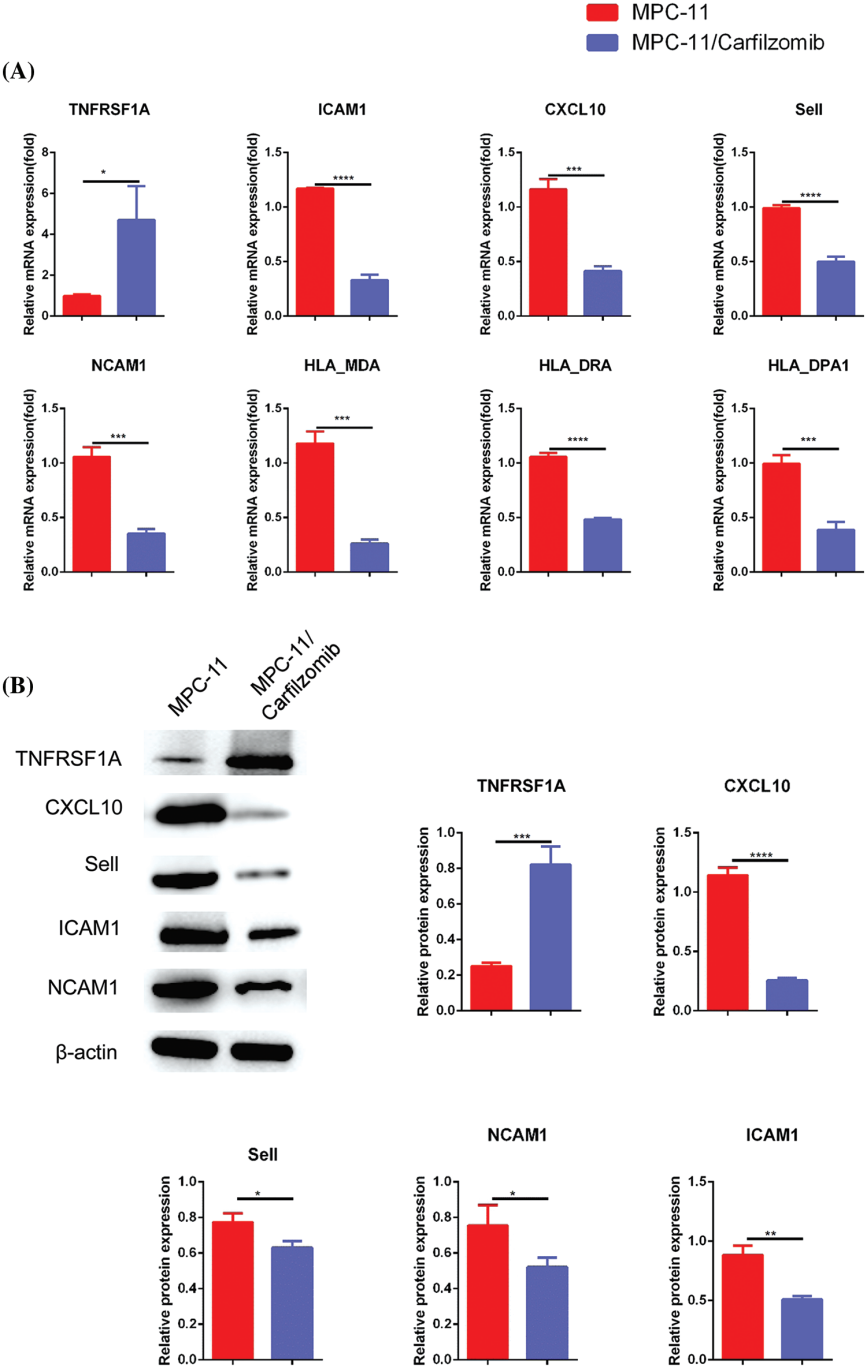
The analyses of *TNFRSF1A*, MHC complex genes and cell adhesion molecules in in carfilzomib-resistant MPC-11 cells and parental MPC-11 cells. (A) qRT-PCR analysis of *TNFRSF1A*, MHC complex genes and cell adhesion molecules in carfilzomib-resistant MPC-11 cells (MPC-11/carfilzomib) and parental MPC-11 cells (MPC-11). (B) Western blot analysis of the protein levels of *TNFRSF1A* and cell adhesion molecules in carfilzomib-resistant MPC-11 cells and parental MPC-11 cells. Data are the summary of 3 independent experiments. *p < 0.05; **p < 0.01; ***p < 0.001; ****p < 0.0001.

Compared with the parental cells, MPC-11/carfilzomib cells exhibited significant upregulation of *TNFRSF1A* at the mRNA level; however, the cell adhesion molecules ICAM1, NCAM1, and SELL; MHC members HLA-MDA, HLA-DRA, and HLA-DPA1; and chemokine CXCL10 were downregulated (Fig. 5A). Furthermore, the protein expression changes in *TNFRSF1A*, cell adhesion molecules, and CXCL10 were confirmed via western blotting (Fig. 5B). Overall, our data suggest that carfilzomib resistance results in *TNFRSF1A* overexpression and the downregulation of cell adhesion molecules and MHC components.

Next, to investigate whether cell adhesion-mediated resistance contributes to carfilzomib resistance of MM cell lines, we co-cultured parental or resistant MPC-11 cells with bone marrow stromal HS-5 cells and analyzed the sensitivity to carfilzomib via flow cytometry. The co-culture of parental MPC-11 cells with HS-5 cells decreased carfilzomib-induced cytotoxicity in MPC-11 cells; this suggests cell adhesion-mediated drug resistance (Suppl. Fig. S1). However, the co-culture of MPC-11/carfilzomib cells with HS-5 cells did not affect carfilzomib resistance. This may be because the decreased expression of cell adhesion molecules did not favor cell adhesion between MPC-11/carfilzomib cells and HS-5 cells. Therefore, the addition of HS-5 cells does not alter carfilzomib resistance.

### TNFRSF1A silencing reverses gene expression and resensitizes MM cells to carfilzomib treatment

**Figure 6:**
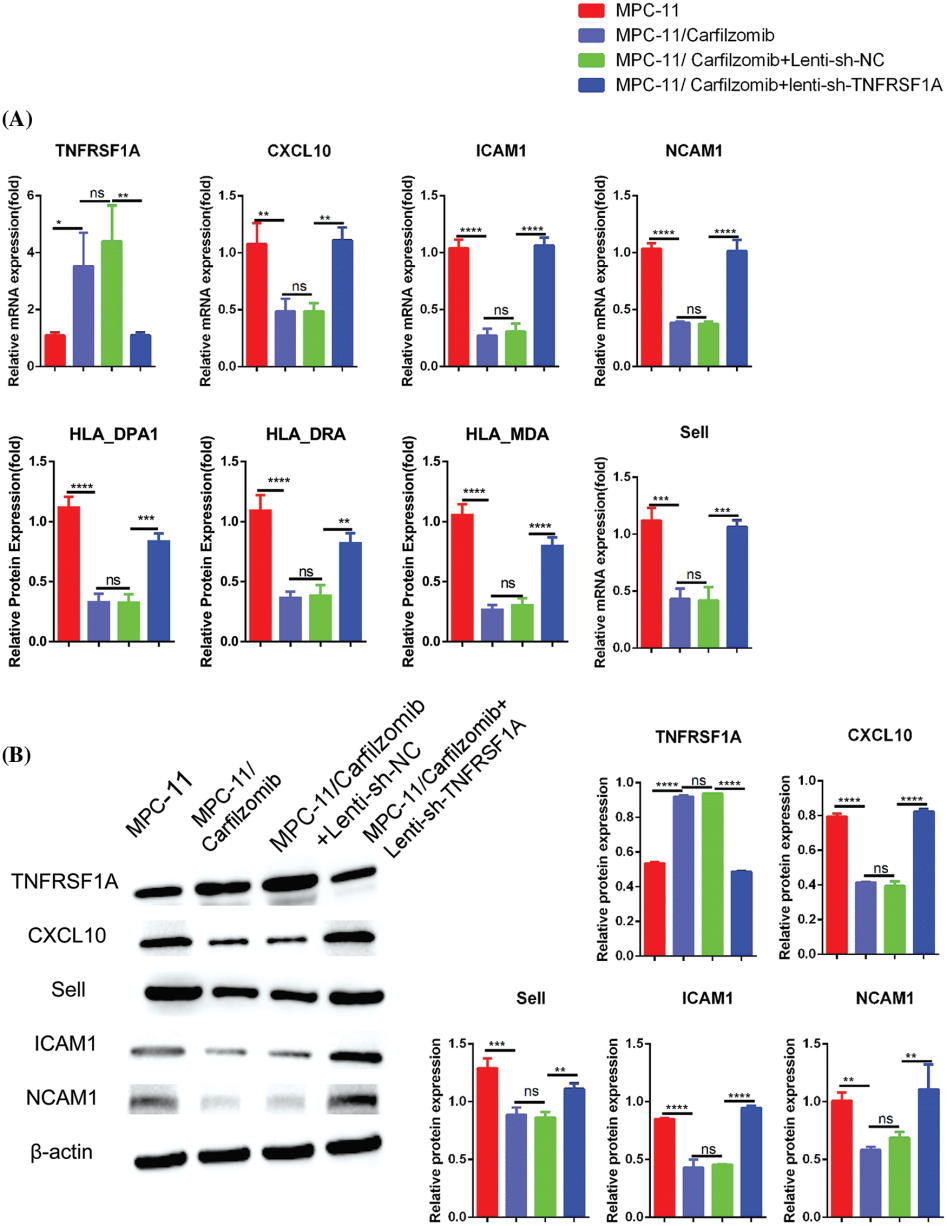
The effect of *TNFRSF1A* silencing on gene expression in carfilzomib-resistant MPC-11 cells. (A) qRT-PCR analysis of *TNFRSF1A* and cell adhesion molecules in parental MPC-11 cells (MPC-11), carfilzomib-resistant MPC-11 cells (MPC-11/carfilzomib), carfilzomib-resistant MPC-11 cells expressing control shRNA (MPC-11/carfilzomib+Lenti-sh-NC) and carfilzomib-resistant MPC-11 cells expressing *TNFRSF1A* shRNA (MPC-11/carfilzomib+sh-*TNFRSF1A*). (B) Western blot analysis of the protein levels of *TNFRSF1A* and cell adhesion molecules in above cells. Data are the summary of 3 independent experiments. *p < 0.05; **p < 0.01; ***p < 0.001; ****p < 0.0001.

**Figure 7:**
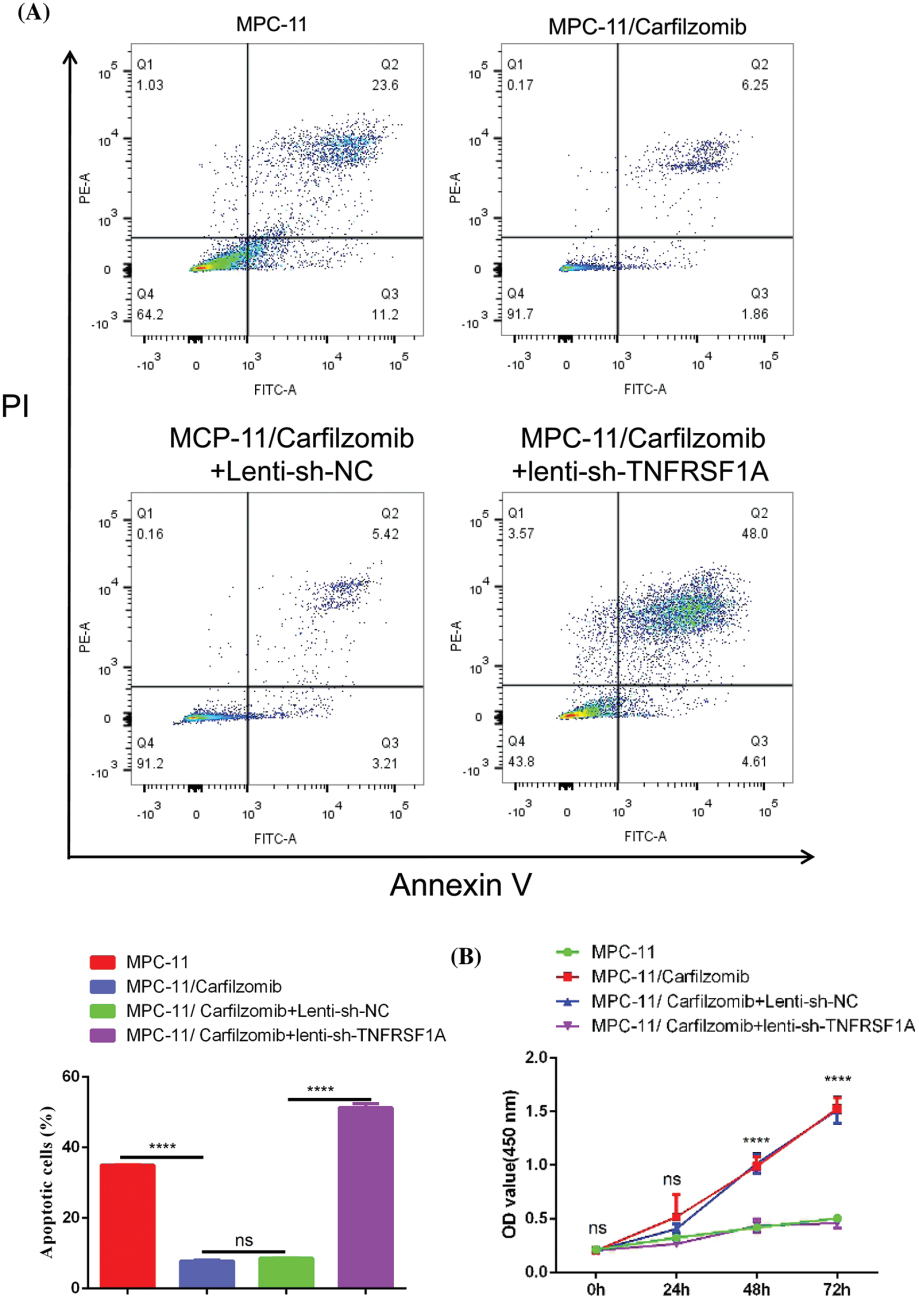
The effect of *TNFRSF1A* silencing on cellular sensitivity towards carfilzomib. (A) Apoptosis detection by flow cytometry in parental MPC-11 cells, carfilzomib-resistant MPC-11 cells, carfilzomib-resistant MPC-11 cells expressing control shRNA and carfilzomib-resistant MPC-11 cells expressing *TNFRSF1A* shRNA after the treatment with 4 nM carfilzomib for 48 h. (B) CCK-8 proliferation assay in above experimental groups in the presence of 4 nM carfilzomib at different time points. Data are the summary of 3 independent experiments. ****p < 0.0001.

*TNFRSF1A* encodes a surface receptor for TNF-α. The binding of TNF-α to the membrane-bound receptor triggers a signaling cascade that can regulate cell survival, apoptosis, and inflammation [21]. However, studies on the role of *TNFRSF1A* in drug resistance in MM cells are lacking. Because *TNFRSF1A* functions as a surface receptor member for signal activation, we hypothesized whether *TNFRSF1A* upregulation facilitates carfilzomib resistance and the associated changes in gene expression. As a result, we used lentivirus to introduce a *TNFRSF1A*-targeting shRNA for *TNFRSF1A* knockdown into MPC-11/carfilzomib cells; *TNFRSF1A* expression was successfully decreased to levels comparable to those in parental MPC-11 cells (Figs. 6A and 6B). On the other hand, *TNFRSF1A* knockdown restored the expression of the cell adhesion molecules ICAM1, NCAM1, and SELL; MHC members; and the chemokine CXCL10 in MPC-11/carfilzomib cells (Figs. 6A and 6B). Furthermore, compared with the sh-NC control, *TNFRSF1A* silencing not only resensitized MPC-11/carfilzomib cells to carfilzomib-induced cell death (Fig. 7A) but also suppressed the proliferation of MPC-11/carfilzomib cells in the presence of carfilzomib (Fig. 7B). Therefore, *TNFRSF1A* overexpression in carfilzomib-resistant MM cells mediates drug resistance toward carfilzomib and decreases the expression of cell adhesion molecules.

Next, to elucidate whether TNF-α signaling affects carfilzomib sensitivity, we treated parental and resistant MPC-11 cells with 10 ng/ml recombinant TNF-α protein and determined the sensitivity of the cells to carfilzomib. We observed that TNF-α treatment increased carfilzomib-induced cell death in parental MPC-11 cells; in contrast, TNF-α treatment did not induce any changes in the sensitivity of MPC-11/carfilzomib cells to carfilzomib (Suppl. Fig. S2A). Furthermore, TNF-α treatment downregulated candidate gene expression in parental cells; however, no changes in gene expression were observed in resistant cells (Suppl. Fig. S2B). Taken together, these results suggest that resistant MPC-11 cells with *TNFRSF1A* overexpression become insensitive to extrinsic TNF-α stimulation owing to the rewiring of the cell signaling pathway after constant *TNFRSF1A* activation.

### TNFRSF1A knockdown reverses carfilzomib resistance of MM cells in the animal model

**Figure 8:**
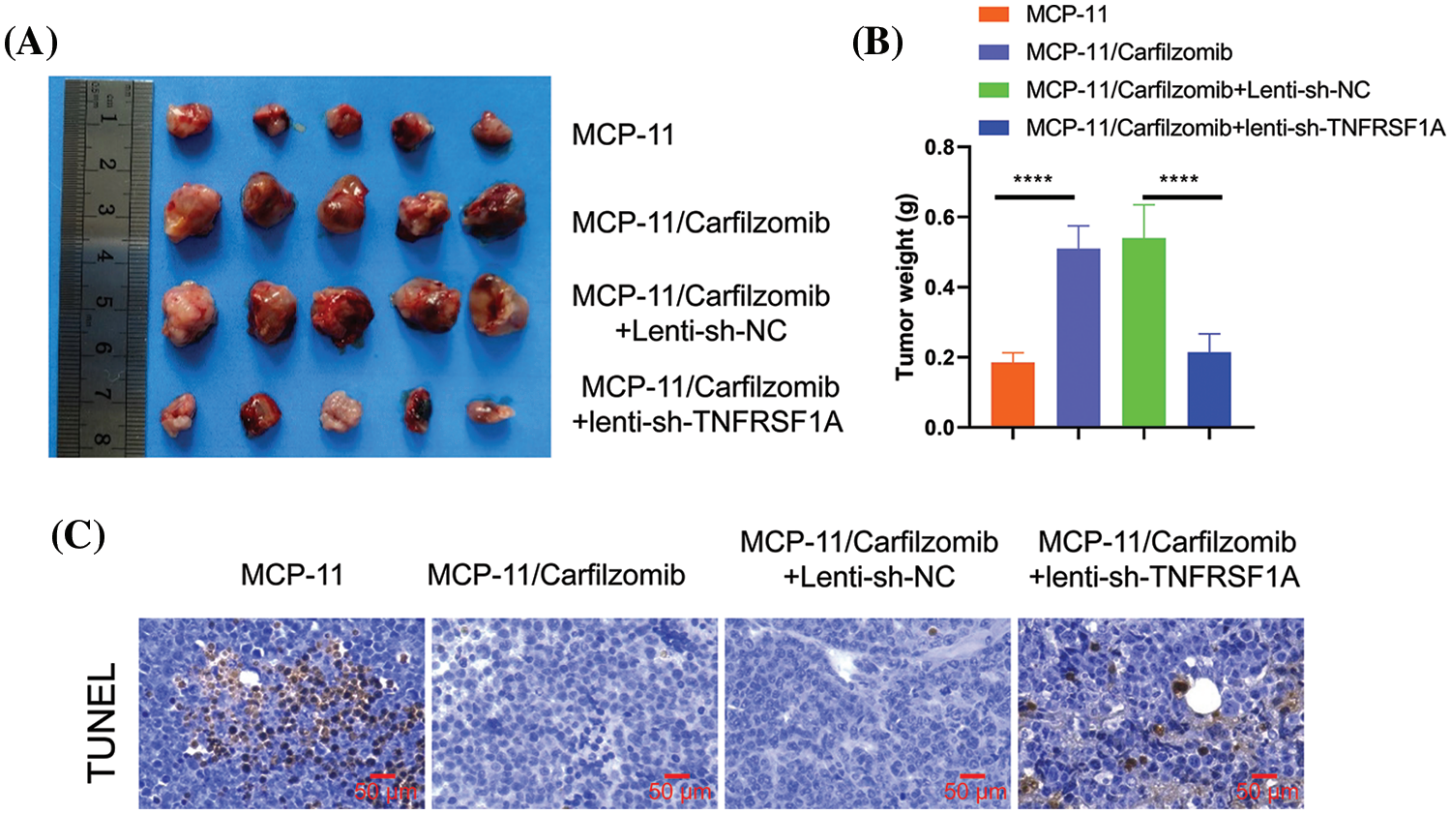
Evaluation of the effect of *TNFRSF1A* silencing on carfilzomib sensitivity towards carfilzomib in animal model. Parental MPC-11 cells, carfilzomib-resistant MPC-11 cells, carfilzomib-resistant MPC-11 cells expressing control shRNA and carfilzomib-resistant MPC-11 cells expressing *TNFRSF1A* shRNA were injected into Balb/C mice (n = 5 in each group), and 3 mg/kg of carfilzomib was administrated by intravenous infusion for two consecutive days within 3 weeks (i.e., Day 1, 2, 8, 9, 15, 16), followed by 12 days of rest (Day 17–28). (A) The images of tumor tissues in each experimental group. (B) Summary of tumor weight at the end of experiment in each group (****p < 0.0001.). (C) TUNEL staining was performed in the tumor tissue sections of each experimental group.

To further validate the role of *TNFRSF1A* in carfilzomib resistance, parental MPC-11 cells, MPC-11/carfilzomib cells, MPC-11/carfilzomib cells with sh-NC, or MPC-11/carfilzomib cells with sh-*TNFRSF1A* were injected into syngeneic mice; all mice were administrated carfilzomib. MPC-11/carfilzomib cells exhibited carfilzomib resistance, as indicated by the larger tumor volume and tumor weight compared with parental MPC-11 cells (Figs. 8A and 8B). On the other hand, MPC-11/carfilzomib cells with *TNFRSF1A* knockdown became more sensitive to carfilzomib treatment compared with those with sh-NC. TUNEL staining revealed that the tumor samples of MPC-11/carfilzomib cells displayed diminished staining signals compared with those of parental MPC-11 cells; in contrast, the tumor samples of MPC-11/carfilzomib cells with *TNFRSF1A* knockdown exhibited a significant increase in TUNEL staining signals (Fig. 8C). Taken together, these findings suggest that *TNFRSF1A* overexpression contributes to carfilzomib resistance of MM cells.

## Discussion

The use of proteasome inhibitors such as carfilzomib has become a mainstay treatment for MM [10,11]. The resulting unfolded protein response and endoplasmic reticulum stress have been proposed to lead to cytotoxicity in MM cells [9]. A key mechanism underlying carfilzomib drug resistance is the upregulation of multidrug resistance proteins [14]. However, the overall picture of the changes in gene expression and pathways after acquiring carfilzomib resistance remains unknown. In the present study, we profiled the published dataset containing parental and carfilzomib-resistant MM cells and observed that cell surface adhesion molecules and multiple MHC components are downregulated in carfilzomib-resistant cells. These results suggest previously underappreciated mechanisms of carfilzomib resistance.

Cell adhesion molecules play a vital role in the invasion and migration of malignant tumor cells during metastasis [22]. In most solid tumors such as breast cancer and glioblastoma, increased expression of cell adhesion molecules contributes to the development of drug resistance [23,24]. Evidence suggests that in MM, treatment with bortezomib can reverse cell adhesion molecule-dependent drug resistance and that this effect may be specific for bortezomib because it has not been observed in other drugs such as vincristine or dexamethasone [25]. This may be owing to the unique mode of action of bortezomib in targeting protein homeostasis. However, the mechanism by which the downregulation of cell adhesion molecules contributes to carfilzomib resistance remains unelucidated. Notably, we observed that cell adhesion molecules may play discrepant roles in myeloma cells when compared with other solid tumors because the upregulation of cell adhesion molecules is often associated with drug resistance in solid tumors [23,24].

The immune microenvironment contributes to the antitumor outcome of several anticancer drugs. The immunomodulatory effect of carfilzomib has been recently discovered. For example, a recent study has reported that carfilzomib can improve immunotherapy by shaping the tumor microenvironment [26]. Furthermore, it exhibits promising efficacy when combined with immunomodulatory drugs for treating refractory MM [27]. However, a study has also reported that the reactivation of the innate immune pathway resensitizes carfilzomib-resistant myeloma cells to carfilzomib treatment [28]. Therefore, this synergistic action with antitumor immunity may constitute a significant fraction of the anticancer effect of carfilzomib. As an evolutionary strategy to survive, myeloma cells can dampen antigen presentation or alter the bone marrow microenvironment to weaken antitumor immunity [17,18]. Our finding that carfilzomib-resistant MM cells exhibit downregulated cell surface adhesion molecules and MHC members is consistent with the hypothesis that drug-resistant myeloma cells escape immune surveillance and re-educate the immune microenvironment [29]. The suppressed expression of cell adhesion molecules can impair the interaction between myeloma and immune cells [30]. On the other hand, the downregulation of MHC members facilitates immune escape by attenuating the presentation of tumor neoantigens [18]. Therefore, we conclude that carfilzomib resistance is accompanied by weakened immune surveillance; this is also consistent with the findings in the immunocompetent mouse model.

The overexpression of *TNFRSF1A* in carfilzomib-resistant MM cells was another key observation in the present study. We observed that the mRNA expression of *TNFRSF1A* was significantly upregulated in carfilzomib-resistant cells, whereas that of the cell adhesion molecules ICAM1, NCAM1, and SELL; MHC members HLA-MDA, HLA-DRA, and HLA-DPA1; and chemokine CXCL10 was downregulated. These data confirm the findings of bioinformatic analyses. Importantly, *TNFRSF1A* knockdown resensitized carfilzomib-resistant cells to carfilzomib as well as reversed the changes in cell adhesion molecules and MHC members. Therefore, *TNFRSF1A* functions as a key regulator of carfilzomib resistance-associated gene expression. Furthermore, we demonstrated that *TNFRSF1A* silencing abrogated carfilzomib resistance in the animal model. Nevertheless, the mechanisms underlying *TNFRSF1A* overexpression in carfilzomib-resistant MM cells should be investigated further. Besides, the downstream signaling pathways mediating *TNFRSF1A*-dependent drug resistance remain unclarified.

As a key member of the TNF-α surface receptor, *TNFRSF1A* orchestrates cellulart signaling pathways to regulate cell survival, apoptosis, and inflammation [21]. TNF-α signaling may play a paradoxical role in cancer development and antitumor immunity. TNF-α, a canonical proinflammatory signaling molecule, activates different immune components to induce antitumor immune responses [31]. Nevertheless, a recent study suggests that the genetic ablation of TNF-α receptors impedes carcinogenesis in a mouse model of skin cancer [32]. Chronic activation of TNF-α signaling can facilitate immune escape by changing the composition and activity of the immune components in tumors [33]. Furthermore, activation of the TNF-α signaling pathway can lead to the upregulation of cell adhesion molecules and changes in the expression of MHC members [34,35]. However, because we observed that resistant MPC-11 cells with *TNFRSF1A* overexpression become inactive to extrinsic TNF-α stimulation, an open question is how constitutive *TNFRSF1A* expression rewires the downstream TNF-α signaling cascade. The answer to this question can shed light on the mechanisms underlying *TNFRSF1A*-dependent regulation of carfilzomib resistance in MM cells.

Notably, several carfilzomib-resistant cell lines such as KMS-11 and KMS-34 can have distinct DEGs; this may be owing to the different protocols used to generate different resistant cell lines [36]. Therefore, future studies should use a standardized protocol to investigate the common determinants of carfilzomib resistance in different MM cell lines and profile primary patient tumor samples with carfilzomib resistance so as to provide more conclusive information to guide therapeutic strategies.

## Conclusions

We noted the upregulation of *TNFRSF1A* and concomitant downregulation of MHC genes and cell adhesion molecules in carfilzomib-resistant MM cells. *TNFRSF1A* knockdown reversed carfilzomib resistance and reactivated the expression of cell adhesion molecules. Furthermore, in immunocompetent mice, *TNFRSF1A* silencing suppressed the tumorigenesis of MM cells, indicating that *TNFRSF1A* leads to carfilzomib resistance by diminishing antitumor immunity. Our findings suggest that *TNFRSF1A* is a plausible target for overcoming carfilzomib drug resistance. Future studies are warranted to elucidate the underlying signaling pathways by which *TNFRSF1A* orchestrates the gene expression changes in MM cells.

## Supplementary Materials

Figure S1 The co-cultured of parental or resistant MPC-11 cells with bone marrow stromal HS-5 cells. Parental MPC-11 cells or the resistant cells were co-cultured with HS-5 cells in the presence of 4 nM carfilzomib for 48 hours. The cell death was analyzed by flow cytometry. Data are the summary of 3 independent experiments. **** *p*<0.0001.

Figure S2 To study whether TNF-α signaling affects carfilzomib sensitivity, parental and resistant MPC-11 cells were treated with 10 ng/ml recombinant TNF-α protein and cultured in the presence of 4 nM carfilzomib. (A). The cell death was analyzed by flow cytometry. Data are the summary of 3 independent experiments. (B). qRT-PCR analyses of cell adhesion molecules in different experimental groups. Data are the summary of 3 independent experiments. * *p*<0.05; ** *p*<0.01; *** *p*<0.001; **** *p*<0.0001.

## Data Availability

The data generated in the present study may be requested from the corresponding author.

## Supplementary Materials

The supplementary material is available online at https://doi.org/10.32604/or.2023.030770.
